# Marriage, Sex, and Hydrocele: An Ethnographic Study on the Effect of Filarial Hydrocele on Conjugal Life and Marriageability from Orissa, India

**DOI:** 10.1371/journal.pntd.0000414

**Published:** 2009-04-21

**Authors:** Bontha V. Babu, Suchismita Mishra, Abhaya N. Nayak

**Affiliations:** 1 Regional Medical Research Centre, Indian Council of Medical Research, Bhubaneswar, India; 2 Social and Behavioural Research Unit, Indian Council of Medical Research, New Delhi, India; Ministry of Health, Burkina Faso

## Abstract

**Background:**

Lymphatic filariasis (LF), a leading cause of permanent and long-term disability, affects 120 million people globally. Hydrocele, one of the chronic manifestations of LF among 27 million people worldwide, causes economic and psychological burdens on patients and their families. The present study explores and describes the impact of hydrocele on sexual and marital life as well as on marriageability of hydrocele patients from rural areas of Orissa, an eastern state of India.

**Methodology/Principal Findings:**

This paper is based on ethnographic data collected through focus group discussions and in-depth interviews with hydrocele patients, wives of hydrocele patients, and other participants from the community. The most worrisome effect of hydrocele for patients and their wives was the inability to have a satisfactory sexual life. The majority of patients (94%) expressed their incapacity during sexual intercourse, and some (87%) reported pain in the scrotum during intercourse. A majority of hydrocele patients' wives (94%) reported dissatisfaction in their sexual life. As a result of sexual dissatisfaction and physical/economic burden, communication has deteriorated between the couples and they are not living happily. This study also highlights the impact on marriageability. The wives of hydrocele patients said that a hydrocele patient is the “last choice” and that girls show reluctance to marry hydrocele patients. In some cases, the patients were persuaded by their wives to remove hydrocele by surgery (hydrocelectomy).

**Conclusions/Significance:**

The objective of the morbidity management arm of the Global Programme to Eliminate LF should be to increase access to hydrocelectomy, as hydrocelectomy is the recommended intervention. Though the study area is covered by the programme, like in other endemic areas, hydrocelectomy has not been emphasised by the national LF elimination programme. The policy makers and programme managers should be sensitised by utilising this type of research finding.

## Author Summary

Lymphatic filariasis, the second leading cause of permanent and long-term disability, affects 120 million people globally. Hydrocele, an accumulation of fluid in the scrotum that causes it to swell, is one of the chronic manifestations of LF among men and there are about 27 million men with hydrocele worldwide. We conducted ethnographic interviews and discussions with patients, women whose husbands have hydrocele, and the general public in a rural community of eastern India. The study describes how hydrocele impacts patients' sexual and marital life. It reveals the most worrisome effect of hydrocele for patients and their wives due to the inability to have a satisfactory sexual life. Patients expressed their incapacity during sexual intercourse. A majority of hydrocele patients' wives reported that their married life became burdened and couples were not living happily. This study also highlights the impact on marriageability, and some women expressed that a hydrocele patient is the “last choice”. In some cases, the patients were persuaded by their wives to remove hydrocele by surgery (hydrocelectomy). Hence, access to hydrocelectomy has to be strengthened under the Global Programme to Eliminate Lymphatic Filariasis, which is operational in several endemic areas in the world. Also, this activity may be integrated with primary healthcare services and interventions of other neglected tropical diseases.

## Introduction

Lymphatic filariasis (LF), the second leading cause of permanent and long-term disability [1], affects 120 million people globally [2]. It is a mosquito-borne parasitic disease caused by *Wuchereria bancrofti*, which accounts for approximately 90% of all LF cases, followed by *Brugia malayi* and *Brugia timoti*. India contributes about 40% of the total global burden of LF. In India, a total of 554 million people are at risk of infection, and there are approximately 21 million people with symptomatic LF and 27 million asymptomatic microfilaria carriers [3]. The manifestations of disease are mostly irreversible and a cause of socioeconomic and psychological problems for patients and often their families [4]–[7]. Hydrocele, an accumulation of fluid in the tunica vaginalis in the scrotum that causes it to swell, is one of the chronic manifestations of LF among men (Figure S1 and Figure S2). There are 26.79 million cases of hydrocele worldwide and 48% of these cases are in India [2]. However, little information is available from India, specifically on disability due to hydrocele, except for a few studies on productivity [6]–[9]. The authors undertook one-year round case-control studies to investigate the economic burden, in terms of treatment costs and loss of work, on people affected with chronic and acute forms of LF in rural communities of Orissa [7],[10]. As part of these studies, epidemiological investigations were also carried out in these communities to identify the cases [11],[12]. The authors' interaction with people during these studies revealed several problems related to marriage and sex due to hydrocele. In addition, it is a neglected area in disease burden research [13]. Thus, it is hypothesised that hydrocele has an impact on marital life and marriageability of affected individuals. The authors have a strong rapport with the communities in this study, given that these were sites for the socioeconomic studies mentioned above. The present study was undertaken using ethnographic methods to explore and describe the impact of hydrocele on marital and sexual life, and on marriageability.

## Methods

### Study Area

The present study was conducted in the Khurda district of Orissa, an eastern state of India. This district is known for endemicity of LF caused by *W. bancrofti*, transmitted by *Culex quinquefasciatus* [11]. The study area is rural in nature and its inhabitants are mostly small farmers and daily wage labourers. The economic opportunities for daily wage labourers are greater in the monsoon period and sparse during the rest of the year.

### Research Methods

This paper is based on ethnographic data, for which the protocols were developed during the LF socioeconomic studies mentioned above [7],[10]. The data for this paper were collected during 2003. Prior to this, the project protocol was approved by the Scientific Advisory Committee (SAC) of the Regional Medical Research Centre, Bhubaneswar. The SAC reviews and approves the research projects for their scientific and ethical merits. A summary of the methodology is presented in Table 1.

**Table 1:** Methodology at a Glance.

Research Method	Study Participants	Number of Participants	Issues Covered
Focus group discussion	Hydrocele patients	Three groups with 6–8 participants in each group	• Problems faced during settlement of marriage
			• Effect of hydrocele during intercourse
			• Dissatisfaction of wife
			• Effect on marriageability
	Community members	Five groups with 6–8 participants in each group (3 groups of men and 2 groups of women)	• Problems faced by hydrocele patients and their families during settlement of marriage
			• Effect of hydrocele on conjugal/sex life
In-depth interview	Hydrocele patients	32	• Satisfaction of his wife to live with him
			• Problems with wife
			• Effect of hydrocele on sexual relations with wife
			• Perceptions on sexual satisfaction of wife
			• Effect on marriageability
	Wives of hydrocele patients	35	• Marital satisfaction
			• Effect of hydrocele on marriageability
			• Satisfaction with her husband being a hydrocele patient
			• Effect of hydrocele on sexual relation with her husband
	Key-informants in the community	9	• Marriage prospects of hydrocele patients
			• Effect of hydrocele on sexual life
**Study location:** 12 villages in Khurda district, state of Orissa, India			
**Study period:** July 2003–December 2003			

Eight focus group discussions were conducted among hydrocele patients and community members separately. Focus groups are widely used to examine people's experience of diseases [14],[15]. They are useful for studying dominant cultural values, such as narratives about sexuality [16],[17], and actively facilitate the discussion of taboo topics. Participants can also provide mutual support in expressing feelings that are common to their group but which they consider to deviate from mainstream culture [16]. Standard guidelines were followed for conducting focus group discussions [16],[18],[19]. Due to the sensitivity of the topic, focus group discussions with community members were conducted separately among men and women. The focus group discussions were conducted in Oriya, the language of the state of Orissa. Question guides were used to ensure that the moderator addressed all the issues to be discussed by the group. Separate guides were prepared for patient groups and the general community. Initially, these guides were prepared in English and translated into Oriya, and all translated guides were reviewed for linguistic reliability and correctness. Later, these guides were piloted with groups similar to the participants' groups, but from villages that were not included in the study, to check appropriateness, clarity, and flow of questions. The duration of focus group discussions varied between 60 and 90 minutes.

To elicit the views and experiences on the impact of hydrocele, in-depth interviews were conducted with hydrocele patients, wives of hydrocele patients, and key-informants in the community. In-depth interviewing offers the respondents the opportunity to express their own ideas and address themes that the researchers may not have anticipated [20]. Key-informants are individuals in the community who possess special knowledge and who are willing to share their knowledge with the researchers. They have access to the culture under study in a way that the researcher does not [18]. The procedures for selecting key-informants and conducting in-depth interviews were based on standard guidelines [18],[21]. All of the interviews were held in Oriya and the duration of these interviews ranged from 30 to 75 minutes. Separate interview guides were made for the three categories of participants. These guides were prepared and finalised in the same manner as the focus group guides.

### Participants

Participants for focus group discussions and one-to-one in-depth interviews were selected by purposive sampling from 12 villages of Khurda district, Orissa. In purposive sampling, researchers choose study sites or informants to represent the range of variation on those characteristics that seem to be meaningful for the topic under study. In this situation, a small number of specially chosen informants can yield valid and generalisable information [18]. Participants were selected from all of the villages to the extent possible. During epidemiological investigations [11], a door-to-door survey was conducted and all individuals in these villages were physically examined for different signs and symptoms of LF. A cohort of 115 patients exclusively with hydrocele was available. These patients were identified for in-depth interviews and focus group discussions, and only those who were married and below 50 years of age were selected. Hydrocele patients who possessed other LF symptoms like lymphoedema were not included. To conduct a focus group discussion with hydrocele patients, six to eight patients from two to three villages were consulted and requested to arrive to a particular place at a particular time. These villages are closely situated within a distance of 2–3 kilometres. Further, they share several common places like agricultural fields, markets, a health centre, etc. Hence, people of these villages were known to each other. Two focus groups consisted of eight patients each and a third group consisted of six patients. The hydrocele patients who were chosen for in-depth interviews were not included in focus groups. The selection of women whose husbands suffer from hydrocele was also made by identifying hydrocele patients from the cohort. These patients were not covered for in-depth interviews and focus group discussions.

Key-informants were also selected from all of the villages, based on the guidelines mentioned above. The key-informants were village/community heads, members of local administrative bodies, and teachers residing in these villages. All of these informants were educated, having at least a school education, and were up to the age of 70 years. For focus groups with community members, participants were selected from within a village. Two groups of women (consisting of six and eight members) and three groups of men (consisting of seven, eight, and eight members) in the age range of 20 to 50 years were selected. Women participants were housewives and the men were farmers and agricultural labourers. Some of the participants did not have any formal education, while others had received formal educations of up to 5 years. During the recruitment of focus group participants, it was ensured that all participants knew each other, and care was taken to maintain homogeneity within each group. Participants having similar socioeconomic characteristics (like education, income, occupation, and caste affiliation) were grouped together.

The purpose of the study was explained and consent to participate in the study was obtained orally from all of the participants, as suggested by the SAC. Oral consent was obtained instead of written consent, as the majority of the study participants were illiterate. The consent of the participants was recorded along with the recording of the interview/discussion. In addition to obtaining consent from each participant, the consent of the community leaders and village heads was obtained to conduct the surveys in their villages and communities. None of the participants declined to particpate in the study.

### Research Team

The researchers and staff who assisted during the conducting of focus groups (SM and ANN, and those listed in Acknowledgments) are aware of local language and culture. The discussion/interview guides were prepared initially in English to ensure that all the issues and objectives of the study were covered comprehensively. The focus group moderators were trained medical anthropologists who were experienced with conducting focus group discussions on LF-related issues. The moderator conducted the focus group discussions, while observers watched and took notes on the discussion, as well as on participants' actions, gestures, and emotions that could not be captured on audiotape. The observers transcribed the focus group discussions. The in-depth interviews were conducted by the same researchers who acted as moderators of the focus group discussions. Focus groups as well as interviews with female and male participants were conducted by female and male staff, respectively. After each discussion/interview, the team along with the primary researcher discussed what had transpired and reviewed the observers' field notes on impressions and observations.

### Data Management

The entire discussion/interview was recorded on audiocassettes. Later, the audiocassettes were played back and transcribed into Oriya with the help of field notes. The Oriya scripts were translated to English and were entered into a personal computer in MS Word as text files [22]. The analysis was done by using ATLAS.ti for Windows 4.1 (Scientific Software Development, Berlin). This computer-based analysis facilitated selecting relevant quotations from the text, coding, annotating, and comparing the quotations. The coding plan was developed based on the issues related to the effect of hydrocele on conjugal life and marriageability. The quotations of each code were retrieved for each category of participants by using a text-based selection option. Thus, by summarising the quotations of each code by category, a matrix was developed to examine the qualitative data.

## Results

### Effect on Sexual Life

Data revealed that hydrocele is always a burden to the patient and to his family. In addition, the most worrisome effect of hydrocele for patients and their spouses was the inability to have a satisfactory sexual life. Of the 32 patients interviewed, 30 patients (93.7%) expressed their frustration due to their incapacity during sexual intercourse. All respondents interviewed except four (87.5%) reported that they get severe pain during intercourse and hence they avoid sex. They also perceived the dissatisfaction of their spouse due to their incapability during sexual intercourse. A young hydrocele patient during an interview said, “*The hydrocele has affected my sexual life. I could not do much … . My wife is now not interested to have sex with me. Seems that she hates me… and advises me to go for hydrocele surgery.*” Another patient narrated, “*My spouse has no complaint, rather she is very cooperative in this matter. However, my sexual desire has declined drastically over the years. I develop an acute pain in the scrotum during and immediately after the intercourse*.” However, in focus group discussions with hydrocele patients, a few participants argued that it has no gross impact on sexual life, though they do experience some pain. However, the groups reached consensus that their condition has influenced their ability to have sexual intercourse.

Of the 35 women whose husbands are hydrocele patients, 33 women (94%) reported dissatisfaction with their sexual life. Some women (25.7%) complained that a failure of erection and penetration occurred during intercourse due to the larger size of the hydrocele scrotum. About half of them said that hydrocele patients have lesser sexual potency than normal men and they thought that hydrocele is responsible for male impotency. Many of these women (68.6%) also reported that their husbands became hesitant to have sex and subsequently lost interest in it. In the voice of the wife of a hydrocele patient, “*When the disease becomes prolonged, the front portion of male genital becomes very small and the scrotum gets unusually large. Eventually that leads to male impotency.*” However, about 6% of these women reported that they did not perceive any problem. This reporting might be due to the following: (i) some men are in an early stage of hydrocele and might not be affected much, and/or (ii) these women may not choose to reveal this private information. A few women could not speak openly, as the issue is sensitive and related to their family. A 28-year-old woman, whose husband is a hydrocele patient, said, “*Yes, there is problem. How there shall be no problem? There is a major problem at the time of intercourse. But I do not like to reveal it to everybody. For instance, when we are served food by our guests, we normally accept it irrespective of whether or not we like that food. For certain things, I cannot tell to my husband, he may feel bad about me. Though we are not happy on bed, I manage it without showing sense of dissatisfaction.*” This study found that the wives of many hydrocele patients persuaded their husbands to get a hydrocelectomy. In one case, it was found that the wife of a hydrocele patient was no longer interested in sleeping with her husband. In another case, as narrated by a woman respondent, a woman had left her husband and gone back to her parents' house out of frustration. During one interview, a woman reported, “*A newly married girl of our village recently left her husband and went back to her parents' house due to this problem. We stay with our husbands because we are old and have children. The younger generation girls are not that much adjustable.*”

The general community also reported similar views about the effect of hydrocele on the sexual life of patients. Of the nine key-informants in the community, eight informants (89%) revealed that the hydrocele patients feel pain during sexual intercourse. They also said that, as the penis of hydrocele patients get shorter, they fail to satisfy their wives during sexual intercourse. These respondents became aware of these issues through casual discussions and gossiping with the affected people and other community members. Hence, these respondents discussed these issues in a generalised manner without referring to a particular person.

### General Marital Life

Concerning marital life, the hydrocele patients revealed several issues. During in-depth interviews, half of the 32 patients agreed that they and their wives are not as happy as they were before the development of hydrocele. When they were asked whether or not both of them live just like others, about 40% of patients said that they are not like others, as their condition hampered their economic situation, because of loss of work capacity, and led to dissatisfaction in their sexual life. About one-third of patients said that their spouses were hesitant and did not like to go out with them. Approximately one-fifth of patients revealed that their wives expressed dislike towards them. The focus groups of patients also revealed that communication had deteriorated between the couples and the quality of the marriage had been affected.

About half of the women (17/35) whose husbands have hydrocele told the interviewers that their husbands' activities were affected adversely due to hydrocele. These respondents reported that patients get severe pain in their genitals (scrotum) and feel too weak to perform hard work. Approximately 60% of women felt sorry for the condition of their husbands. About one-third of the respondents reported that the disease has affected the economic condition of their family. Some of these respondents (11.4%) reported that their husbands drink alcohol to get rid of the pain and other problems associated with hydrocele. A few women said that they frequently quarrel because of the disease and their relations are deteriorating. The following statement of a 40-year-old woman reveals the agony of such women: “*My husband has a big hydrocele. He is not able to move and work freely. He feels ashamed to make his appearance in public places. He is now not even fit for conjugal life*.”

The community members also perceived the problem of hydrocele and revealed that the disease is affecting people physically and psychologically. They mentioned several instances in which the deterioration of relations between wife and husband and disturbances in conjugal life had occurred.

### Effect on Marriageability

The hydrocele patients also revealed the impact of hydrocele on marriageability. They said that one should remove it by surgery before going for marriage proposals. All patients agreed that it is difficult to get a bride for a hydrocele patient. Some patients in focus groups said that some men remained unmarried due to their condition, but this was denied by a few patients. However, all focus groups of patients agreed that it is problem for young men to get married, as the patient cannot work or even walk properly. In the present study, the hydrocele patients anticipated problems in getting their children married due to their disease. This disease is considered hereditary and people think that the diseases get transmitted to the next generation. When their sons suffer from hydrocele, it then becomes increasingly difficult for the parents to obtain spouses for their sons.

The women whose husbands have hydrocele said that the hydrocele patient is the “last choice” and that some girls are reluctant to marry hydrocele patients. A woman who is the wife of a hydrocele patient reported, “*Had I know about the hydrocele of my husband, I could have refused to marry him.*” About half of the women whose husbands were suffering from hydrocele said that their husbands had this disease before marriage. Approximately two-thirds of them alleged that the husbands' families had not disclosed their husbands' disease prior to the marriage, and these women expressed the feeling of having been deceived. Some women also expressed their inability to choose a bridegroom due to their economic situation and family customs. The women were asked a hypothetical question querying whether they think getting a spouse for a hydrocele patient is difficult. More than half of these participants said that it is difficult to get spouse for a hydrocele patient. Specifically, if a girl knows beforehand about the disease of the bridegroom, she may not accept that proposal. However, some women respondents (34%) opined that it is not a problem, as hydrocele can be removed by surgery.

The community members also felt similarly and said that people do not prefer to give their daughters to a hydrocele patient. Therefore, the hydrocele patients marry with little or no dowry under a compulsive situation. Often they marry girls from lower socioeconomic strata. One person explained, “*It is difficult for a filariasis patient to get a girl by choice for marriage. Normally he gets married to a girl of lower economic status. … Parents offer their daughter to such a patient only under compulsive situations.*”

## Discussion

In LF-endemic areas, hydrocele develops from asymptomatic infection through acute clinical manifestations. There are about 73 million people with LF infection worldwide and men in this group are at the risk of developing hydrocele, in addition to 27 million existing hydrocele patients [2]. The peak incidence of noticeable hydrocele seems to occur in early adulthood, between the age of 19–34 years [2] (Figure S1). It is demonstrated through several studies that hydrocele has an immense impact on economic activities and productivity [6],[7],[9],[23],[24] and quality of life [25],[26]. Addiss and Brady [27] reported that hydrocele patients reported both “enacted stigma” and “felt stigma”, based on studies in some endemic areas [28],[29]. Some patients often described themselves as frustrated, losing hope, and even suicidal [5],[30],[31].

The present study highlights the impact of hydrocele on conjugal life and relations between hydrocele patients and their wives. In addition, the paper describes how hydrocele impacts the marriageability of patients and their sons. Hydrocele affects men during the prime age when they pursue social and family goals. Women married to hydrocele patients were “silent sufferers” of their husbands' disease. The dissatisfaction of the patient and his wife leads to the deterioration of the marital relationship. However, in a society like rural India, the institution of marriage is strong and women usually do not separate for reasons like sexual dissatisfaction. The family and society do not appreciate and support such women, in addition to the existence of a strong community sanction against divorce and even temporary separation. However, an incidence of temporary separation due to this problem was reported by a woman participant of this study.

Similar findings were reported from other endemic areas. Dreyer and her colleagues found problems among clinic-based patients from Brazil such as marriages devoid of physical and sexual intimacy, a “conspiracy of silence” that includes both the patient and his wife, and profound shame and suicidal thoughts among men with hydrocele [5]. In Ghana, unmarried men found it difficult to find a spouse of their choice, and various degrees of sexual dysfunction were reported amongst married men [30]. This study associated sexual dysfunction with the size of the hydrocele. However, in the present study, no such attempt to find an association with the size of hydrocele was made due to lack of sufficient data. A similar study from another endemic area of Ghana reported the inability of hydrocele patients to have satisfactory sexual intercourse [28]. In addition, hydrocele prevented patients from getting a marriage partner, and there were a few cases of divorce due to hydrocele [28]. Another study, based on the extended Euro quality of life scale among South Indian hydrocele patients, reported that hydrocele adversely affected the patients' sexual functioning and caused moderate problems with anxiety/depression [32]. Though these studies reported sexual dysfunction and its effect on married life, the strength of the present study is that it could capture the feelings of the wives of hydrocele patients. Many wives persuaded their husbands to remove hydrocele by surgery. Women in endemic areas may have an important role in advocating for better access to hydrocelectomy, at least on a household level. However, it is felt that these issues, specifically the feelings of the women, should be understood with gender perspectives.

There are methodological limitations in this study, as is usual with this type of research design and methods. The topics of discussion and interviews are sensitive, and participants may not express their views openly, as they think that their responses may damage their reputation or their family. Sometimes, in this type of research, participants may also report the behaviour that is believed to be consistent with their culture, rather than the actual behaviour [33]. In focus groups, some participants were inactive and did not reveal much in the discussion, while some others were active. Though that is a limitation, it has been managed by the trained moderators.

It is clear that men with hydrocele need psychological and social support, as opined by Dreyer and her colleagues [5]. As there is a strong feeling of shame and embarrassment among hydrocele patients, the problem of sexual disability is usually not acknowledged unless the patients are specifically probed by health care staff. The desire of some of the hydrocele patients as well as their wives was to have surgery to remove the hydrocele (hydrocelectomy). In addition, a majority of these people were aware of the remedy of hydrocele through the surgery [34]. However, most hydrocele patients have not had a hydrocelectomy due to the costs involved, loss of working days/wages during hospitalisation and recuperation after surgery, and lack of a surgical facility in rural public health institutions. The surgical facilities for hydrocelectomy are available in private hospitals in urban areas, and these hospitals charge more than US$100 for surgery and a bed. In addition, the patient has to bear the other expenditures like medicines, food, travel, etc. This expenditure, along with the loss of work and wage of the patient as well as the escort, prevents patients from accessing surgery for the cure of hydrocele.

This study area is covered by the Global Programme to Eliminate LF. The alleviation of disability and control of morbidity among LF patients is the second arm of the programme [35]. This arm has not received much attention and therefore lags behind the first arm of the programme, i.e., interruption of LF infection through mass drug administration (MDA). It is evident by the fact that 48 of the 83 endemic countries had implemented MDA by the end of 2007, whereas only 27 of the 48 countries that implemented MDA have initiated morbidity management activities [36]. Even in those areas where morbidity management activities are conducted, more emphasis is given to the management of lymphedema, rather than to the repair of hydrocele. This could be due to several reasons, including lack of resources at health institutions of an implementation level and lack of adequate information on the burden and impact of hydrocele. The policy makers and programme managers should be sensitised by using research findings on the burden and impact of hydrocele. The objective of any LF morbidity management programme should be to increase access to hydrocelectomy. One of the initial activities of the programme should be to detect hydrocele cases using existing community-based surveys, such as enumeration during MDA. In addition, a house-to-house morbidity census may be conducted to acquire a better understanding of hydrocele burden. Individuals with hydrocele should be referred to a facility for surgery, if necessary. Mass hydrocelectomy camps may be feasible initially to reduce the burden of hydrocele in high LF-endemic areas. In a study from Ghana, patients reported that within 3 to 6 months of the post-surgery period, they had experienced a significant improvement in self-esteem, sexual function, and work capacity, and they participated more in community activities [28].

Because MDA may be used as a primary prevention measure for disabilities caused by LF, opportunities for synergy between MDA and disability management and prevention activities need to be explored [37]. Endemic countries have become convinced of the benefits of the programme and real progress in arresting transmission has been reported from countries that commenced MDA early [38]. Also, an unpredicted outcome of MDA, i.e., reduction of incidence of hydrocele following MDA, has been reported from Papua New Guinea [39] and India [40]. However, a common finding was that even after an aggressive control programme to arrest the transmission of infection, chronic manifestations such as hydrocele have persisted for decades [41]. Hence, the programme's two arms should go hand in hand. There is a need to incorporate LF disability management and prevention into primary health care services, which are well established in many areas. Also, these activities may be integrated with health interventions of other neglected tropical diseases. The integration among these programmes helps improve both efficiency and effectiveness [42]. Recently, there has been significant discussion on potential challenges, opportunities, and estimated potential benefits including cost savings [42]. The momentum of the Global Programme must be sustained to remove the impediments that prevent hydrocele patients from leading a decent life and to stop generation of new hydrocele patients.

## Supporting Information

Figure S1 This photograph is of a teenager from an eastern Indian village suffering from filarial hydrocele. He is one of the thousands of victims of the disease who were burdened with shame and suicidal thoughts. Their prospects of getting married were diminished due to their condition, in addition to its impact on work and productivity.
(0.07 MB TIF)

Figure S2 This photograph is of a person from a filarial-endemic area of eastern India. He was suffering from both chronic forms of lymphatic filariasis, i.e., lymphedema and hydrocele. These conditions adversely affect patients' status in the community and cause agony and depression among patients and their families.
(0.12 MB TIF)
