# Supplementary figures and images for: Marriage, Sex, and Hydrocele: An Ethnographic Study on the Effect of Filarial Hydrocele on Conjugal Life and Marriageability from Orissa, India
Source: PLoS Negl Trop Dis. 2009 Apr 21;3(4):e414. doi: 10.1371/journal.pntd.0000414 (PMC2666802; doi:10.1371/journal.pntd.0000414)

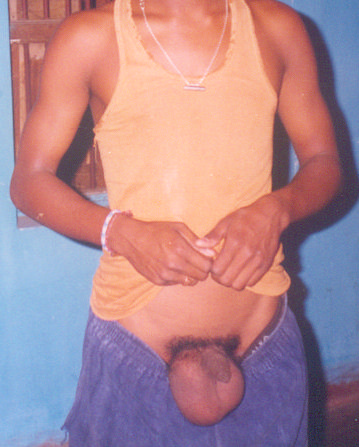

Supplement: Figure S1 — This photograph is of a teenager from an eastern Indian village suffering from filarial hydrocele. He is one of the thousands of victims of the disease who were burdened with shame and suicidal thoughts. Their prospects of getting married were diminished due to their condition, in addition to its impact on work and productivity. (0.07 MB TIF) [file pntd.0000414.s001.tif]

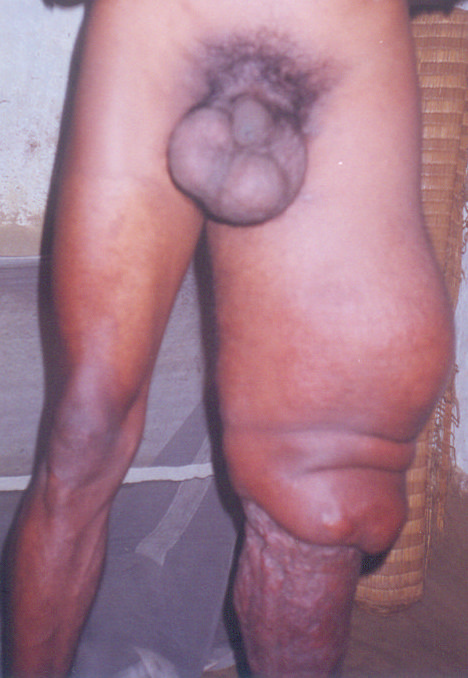

Supplement: Figure S2 — This photograph is of a person from a filarial-endemic area of eastern India. He was suffering from both chronic forms of lymphatic filariasis, i.e., lymphedema and hydrocele. These conditions adversely affect patients' status in the community and cause agony and depression among patients and their families. (0.12 MB TIF) [file pntd.0000414.s002.tif]
